# The evaluation of iron deficiency and anemia in male blood donors with other related factors

**DOI:** 10.4103/0973-6247.67032

**Published:** 2010-07

**Authors:** Vahid Yousefinejad, Nazila Darvishi, Masoumeh Arabzadeh, Masoumeh Soori, Mahtab Magsudlu, Madjid Shafiayan

**Affiliations:** *Deputy of Research, Kurdistan University of Medical Sciences*; 1 *Sanandaj District Health Center, Kurdistan University of Medical Sciences*; 2 *Kurdistan Organization of Blood Transfusion*; 3 *Iranian Organization of Blood Transfusion -Research Center*; 4 *Bachelor of Nursing, Kurdistan University of Medical Sciences*

**Keywords:** Blood donors, iran, iron deficiency anemia, iron deficiency, males

## Abstract

**Aims and Background:**

Iron deficiency is one of the most common nutritional disorders worldwide and blood donation may cause iron depletion. Limited studies with large sample size have been done on male donors. The aim of this study is to determine the prevalence of iron deficiency and iron deficiency anemia among male donors in the Kurdistan Organization of Blood Transfusion in Iran.

**Materials and Methods:**

This was a cross-sectional study. Sample size was 1184 blood donors selected by systematic random sampling. Hemoglobin, serum iron, serum ferritin, total iron banding capacity (TIBC) and transferin saturation were measured in donors. Iron depletion, lack of iron stores, iron deficiency, iron deficiency anemia and anemia were evaluated among them. Data was analyzed with SPSS software and X^2^, one-way ANOVA, and LSD test.

**Results:**

Iron deficiency, anemia, iron deficiency anemia, iron depletion and lack of iron resources were seen in 2.3, 4.08, 2.14, 22.76 and 4.66 percent respectively. There was a significant relationship of iron deficiency and iron deficiency anemia with instances of donation and interval from last donation (*P* < 0.05). A significant relationship was seen between iron deficiency and iron deficiency anemia among blood donors with more than ten times blood donation (*P* < 0.05).

**Conclusions:**

This study showed regular male donors require especial attention. Therefore, serum ferritin is recommended as a more adequate index to use for iron deficiency screening and planning purposes for iron supplementation among them.

## Introduction

Iron deficiency is the most common nutritional disorder in the world,[1] and chronic iron deficiency is a well-recognized complication of regular blood donation.[2] Previous data for iron deficiency among regular male donors in several surveys had been different (5.5-28 percent).[1,3,4] A majority of previous studies have emphasized on iron deficiency among female donors.[1,2,5]

A majority of surveys have mentioned the relationship between prevalence of iron deficiency anemia and increase in instances of blood donation.[2–10] Also, a significant relationship was seen between the mean of serum ferritin and instances of blood donation, and this suggests estimation of serum ferritin to check preclinical iron deficiency among donors.[2,6–9]

But in several countries like Iran, checking serum ferritin is not recommended for evaluation of serum iron status among donors, and there is no definite and special protocol for prevention of iron deficiency especially in male donors. Restriction in number of samples, especially among male specimens in a majority of previous studies is the reason for limitation of these surveys’ results. It seemed helpful for this hypothesis to do a study with a large sample volume among male donors. This study was carried out with an aim to assess iron deficiency status and iron deficiency anemia among male donors and their related factors as a precursor for future studies in planning a project to prevent iron deficiency and IDA among male donors in the Kurdistan Organization of Blood Transfusion of Iran in 2007.

## Materials and Methods

This study was a descriptive cross-sectional study. The statistical universe included all male donors in the Kurdistan Organization of Blood Transfusion of Iran in 2007. The sample size was 1184 persons selected by systematic random sampling, and finally 1181 records were analyzed. Note that there are nearly 800-850 male blood donors coming to our center in a month and we considered five months as a period for gathering samples, therefore out of an estimated 4200 donors in this period, after selection of first sample among ten initial donors using a random number table, the remaining samples were chosen at four intervals. Inclusion criteria contained: confirmed donation after examination and rapid hemoglobin (Hb) test, without anemia in past history. In each questionnaire, the selected donors were asked specific questions i.e., time of first donation, total number of lifetime donations, number of donations within previous one year, interval between donations, date of last/previous donation, and history of anemia. After taking participants’ consent, four cc clotted blood, and two cc blood samples were taken, including EDTA in order to do tests (CBC, Iron, TIBC, ferritin). The percent of transferin saturation (TS) was calculated in proportion to iron/TIBC.

CBC test has been done using a full automatic cell counter instrument (Medonic-CA 530) made in Sweden. The ferritin test was performed by Elisa instrument Stat Fax 2100 made in the United States. Pishtazteb kit was used to measure ferritin as approved by the Iranian Ministry of Health. Parsazmoon kit, approved by the Iranian Ministry of Health and biochemical reader photometer Stat Fax (DA-450) instrument made in the United States were used to measure serum iron. For the purposes of this survey, iron depletion, lack of iron stores, iron deficiency, and iron deficiency anemia are defined below.

Iron depletion: serum ferritin less than 20 μgr/dl.[1,11]

Lack of iron stores: serum ferritin less than 12 μgr/dl.

Iron Deficiency: serum ferritin less than 12 μgr/dl and transferin saturation percentage less than 15. Iron deficiency anemia: serum ferritin less than 12 μgr/dl, transferin saturation percentage less than 15, and Hb less than 14 mg/dl.[1,12] Collected data was analyzed with SPSS software using x^2^ test and one-way ANOVA test, and to compare among groups LSD test was used.

## Results

The study’s universe was 1181 people, of which 254 (21.5%) were first time donors, 413 (35%) were recorded ones (interval from last donation longer than one year), and 514 (43.5%) were regular ones (interval from last donation shorter than one year with optimum four donations per year). The mean sample age was 35.34±11.37 years (17.00-63.00). The mean of instances of donation among recorded donors was 5.58±6.90 and among regular donors 12.13±15.15. The mean blood donation interval among recorded donors, 85.29±65.75 months, and among regular donors was 76.16±75.54 months.

Iron deficiency was seen among 12 people (one percent) of samples all of whom were regular donors (100%) (*P* = 0.000). Twelve regular donors (2.3%) had iron deficiency. Eight recorded donors (2.9%) with ten instances of donation and more had iron deficiency, meanwhile among the group of donors with nine instances of donation and less, iron deficiency rate was 0.4% (4 people) (*P* = 0.000).

Anemia was seen among 33 (2.8%) samples, 21 (63.63%) of whom were regular donors and 4.08% of regular donors had anemia. Anemia was seen in 15 persons (3%) donors with 2-9 instances of donation, four (2.8%) donors with 10-19 instances of donation, and eight (5.9%) donors with 20 instances of donation and more (*P* = 0.07). Iron deficiency anemia was seen among 11 (0.9%) samples all from regular donors (100%) (*P* = 0.001), and 2.14% of regular donors had IDA. Seven of recorded donors (2.5%) with ten instances of donation and more had iron deficiency anemia, meanwhile in the group of nine and less instances of donation, the iron deficiency anemia rate was 0.4% (4 people) (*P* = 0.002). Iron depletion was seen among 167 (14.1%) samples, and among this number 117 (70.05%) were regular donors (*P* = 0.000). This means that 22.76% of regular donors had iron depletion. Lack of iron stores was seen among 34 (2.9%) samples, and 24 (70.6%) of them were regular donors (P = 0.005), that is to say 4.66% regular donors had a lack of iron stores. A significant relationship was not seen between blood donation intervals and iron deficiency, anemia, and iron deficiency anemia among donors.

There was a significant relationship between the last donation period, iron deficiency, and iron deficiency anemia among donors (P = 0.02), therefore there was a higher percentage of iron deficiency and iron deficiency anemia among donors with record of 12 months ago and less in comparison to the group with longer than 13 months. There was no significant relationship between age groups and iron deficiency, anemia, and iron deficiency anemia either.

Table 1 shows blood indexes based on individual types of donation. There was a significant relationship between Hb rates, serum ferritin, TIBC, serum iron, transferin saturation, and increasing instances of donation (*P* < 0.05) [Table 2]. There was no significant relationship between Hb rate, MCV, serum ferritin, TIBC, serum iron, and transferin saturation with donation intervals (*P* > 0.05) [Table 3]. There was no significant relationship between Hb rate, MCV, TIBC, and interval from last donation (*P* > 0.05). But there was a significant relationship between serum ferritin, serum iron, transferin saturation, and interval from last donation (*P* < 0.05).

**Table 1:** The mean of blood indexes studied among samples based on donation type in the Kurdistan Organization of Blood Transfusion station in 2007.

Transferin Saturation (mean ± std d)	Serum Ferritin (mean ± std d)	Serum Iron (mean ± std d)	TIBC (mean ± std d)	MCV (mean ± std d)	Hb (mean ± std d)	(n)	Blood index Donation Type
31.54 ± 7.96	59.07 ± 41.28	96.27 ± 16.73	310.67 ± 24.97	84.68 ± 5.96	14.06 ± 0.99	254	First time
31.72 ± 7.73	62.90 ± 41.59	97.17 ± 17.60	310.79 ± 22.96	85.11 ± 5.70	14.06 ± 1.05	413	Recorded
29.94 ± 7.06	40.67 ± 28.47	92.72 ± 16.64	314.50 ± 24.49	83.85 ± 6.36	13.91 ± 1.08	514	Regular
30.91 ± 7.54	52.40 ± 37.81	95.04 ± 17.11	312.38 ± 24.12	84.47 ± 6.07	14.00 ± 1.05	1181	Total

In a one-way AN OVA test, there was a significant difference between Hb figure (*P* = 0.05), MCV (*P* = 0.006), TIBC (*P* = 0.03), serum iron (*P* = 0.000), serum ferritin (*P* = 0.000), and transferin saturation (*P* = 0.001) among donor based on type of donation.

**Table 2:** The mean of blood indexes studied among samples based on instances of donation in the Kurdistan Organization of Blood Transfusion station in 2007.

Transferin Saturation (mean ± std d)	Serum Ferritin (mean ± std d)	Serum Iron (mean ± std d)	TIBC (mean ± std d)	MCV (mean ± std d)	Hb (mean ± std d)	(n)	Blood index Instances of Donation
31.58±7.95	59.23±41.29	96.36±16.69	310.56±24.95	84.64±5.94	14.07±0.99	253	First records
32.04±7.23	56.94±34.72	97.89±15.95	309.37±20.70	85.64±5.09	14.14±0.96	149	once
31.64±7.60	56.30±41.55	96.79±17.84	310.30±23.08	84.41±6.29	14.08±1.08	360	2‐5
29.86±7.53	47.94±35.89	92.68±17.33	315.58±24.73	83.72±6.27	13.86±1.04	207	6‐10
29.44±6.90	40.46±26.75	91.49±16.43	315.35±25.02	84.01±6.31	13.86±1.15	134	11‐20
28.47±6.47	35.99±21.00	89.65±15.16	320.02±26.58	84.70±6.03	13.73±1.08	78	21 and more
30.91±7.54	52.40±37.81	95.04±17.11	312.38±24.12	84.47±6.07	14.00±1.05	1181	Total

In a one-way ANOVA test, there was a significant difference between Hb (*P* = 0.004). TIBC (*P* = 0.001), serum iron (*P* = 0.000), serum ferritin (*P* = 0.000), and transferin saturation (*P*= 0.000) among donors based on instances of donation. But there was no significant relationship between MCV figures and instances of donation (*P*=0.08).

**Table 3:** The mean of blood indexes studied among samples based on donation intervals in the Kurdistan Organization of Blood Transfusion station in 2007.

Transferin Saturation (mean ± std d)	Serum Ferritin (mean ± std d)	Serum Iron (mean ± std d)	TIBC (mean ± std d)	MCV (mean ± std d)	Hb (mean ± std d)	(n)	Blood index Donation length of time
31.39±6.06	45.67±32.66	96.26±14.27	309.43±18.38	85.36±5.97	14.18±1.01	110	One year and less
30.05±7.11	48.49±30.44	93.01±16.35	314.42±25.17	84.58±5.79	14.03±1.00	149	13-24 months
31.01±8.17	49.76±35.48	95.57±19.04	313.48±24.52	83.93±6.49	14.02±1.11	416	25-60 months
30.55±7.13	52.63±39.64	94.12±16.47	312.61±24.02	84.59±5.90	13.89±1.07	506	61 months and more
30.91±7.54	52.40±37.81	95.04±17.11	312.38±24.12	84.47±6.07	14.00±1.05	1181	Total

In a one-way ANOVA test, there was no significant difference between Hb (*P* = 0.09). MCV (*P* = 0.2), TIBC (*P* = 0.4). serum iron (*P* = 0.3), serum ferritin (*P* = 0.3), and transferin saturation (*P*= 0.5) among donors based on donation intervals. But an LSD test and couple comparison among groups showed donors with record of one year and less have significantly higher Hb rates than ones with records of 2-5 years (*P* = 0.05).

## Discussion

Iron deficiency is the most common nutritional disorder in the world, and blood donation is able to cause iron depletion.[1] There have been limited studies with large sample size focusing on donors, especially male donors aimed at evaluating iron deficiency indexes.[7] Given the definitions, 2.3% of regular donors had iron deficiency compared with a survey in Brazil that recorded 5.5% with iron deficiency, with a smaller sample size and considering ferritin less than 15 as an index.[4] In comparison with Javadzadeh and colleagues, who reported 28% of iron deficiency in 199 male donors,[1] our obtained figures are lower in the present study. Despite the similarity of definition in these two studies, the observed difference may be caused by small sample size effect in that study, nutritional status of different regions of Iran and also, instances of blood donation in regular donors in the two surveys.

Lack of iron resources was seen among 4.66% of regular donors. In a survey by Milman and colleagues among 1348 male donors, 6% of samples revealed ferritin under 15,[7] compared with our results (2.9%); with ferritin under 15 considered as lack of iron reserves index in our study, this percentage rises to 5.2%, and compares with that study. However, in comparison with a study in India that reported ferritin less than 15 among 21% of samples, our result has lower figures.[2]

The ferritin is lower than 15% among 3.3% of male donors in a survey in Denmark which compares with our study’s results.[8]

Iron deficiency anemia was seen among 2.14% of regular donors in comparison to another survey in Iran that observed iron deficiency anemia among 16% of regular male donors,[1] a quite lower figure, but it is pretty comparable to a survey in Denmark, with ferritin less than 15, Hb lower than 12.9 and also ferritin less than 15, Hb lower than 13.7, rate of iron deficiency anemia among males recorded at 0.26% and 0.5% respectively.[8]

Iron depletion was also seen among 22.76% of regular donors comparable with a study in Thailand that reported 21.21% iron depletion among samples.[6] But our result had lower figures compared with a study in Poland that reported iron depletion of 49.7%.[13] Considering more than ten instances of donation, iron depletion increases to 31.77% in the present survey and it is nearly comparable with the Poland survey. Iron depletion in regular male donors recorded in Javadzadeh’s study[1] has higher figures than our results. Per our results, 60.3% of regular donors have records lower than ten instances of donation, in other words a great percentage of regular samples in this study were young regular donors. This point should be considered to justify the lower percentage of iron deficiency and iron deficiency anemia in this survey in comparison to some similar studies.[1]

In the present study the Hb average among first time donors, recorded and regular were respectively: 14.06±0.99, 14.06±1.05 and 13.91±1.08 comparing with a study in Malaysia whose observed means were respectively 15.56±1.48, 15.12±1.44 and 14.95±1.08.[9] From the point of view of Hb falling among donors the studies are comparable, but indicate higher total Hb rates among donors in that study.

Comparing the ferritin mean among first time, recorded and regular donors in our study with studies performed in Thailand[6] and Malaysia,[9] indicates iron reserves decreasing among donors in our country since first time donation, leading to accelerating the process of iron deficiency creation among donors. But these means were relatively higher than mean ferritin in a survey in India.[2] [Table 4]

**Table 4:** Published comparative data on serum ferritin level in male blood donors

Regular Donor (mean ± std d)	Recorded Donor (mean ± std d)	First Donor (mean ± std d)	No. samples	References
62.0±39.78	114.12±66.97	90.7±66.63	211	Norashikin *et al*.[9]
33.6±37.03	--------	55.6±51.54	73	Mittal *et al*.[2]
52.72	--------	161.12	82	Tardtong *et al*.[6]
40.67±28.47	62.90±41.59	59.07±41.28	1181	Present study

The donors recorded with more than five instances of donation have lower Hb rates (*P* = 0.004) than ones recorded with five occasions and less, and this inverted relation of a rise in instances of donation and fall in Hb in the present study compares with previous studies.[1,9,10]

There was a significant relationship between serum ferritin rates, serum iron, TIBC, and rising instances of blood donation (*P* < 0.001). This completely correlates with the results of studies done in Malaysia,[9] Tehran University of Medical Sciences,[10] Javadzadeh and colleagues.[1]

In this study the donors with more than five instances of donation have significantly lower serum ferritin than ones with five instances of donation and less (*P* = 0.01), comparing with the results of two previous studies performed in Iran[1,10] while a study in Pakistan mentioned the obvious relationship between four and more instances of donation in the past two years and iron deficiency.[14] Therefore it seems more primary evaluations before blood donation are essential for regular male donors from the fifth donation onwards with not just attention to Hb status; usage of iron supplements in this group should be assessed and this matter had been emphasized in previous studies as well.[1,7,8,10,13,15–18]

There was also a significant relationship between existence of iron deficiency and iron deficiency anemia with instances of blood donation with considered definition in this study (P < 0.005). Therefore, the rates of iron deficiency and iron deficiency anemia were significantly higher among donors with more than ten instances of donation. This significant difference in the majority of iron deficiency indexes among individuals with more than five instances of donation in comparison to ones with less gives rise to a hypothesis that indexes change from fifth donation and above, and creation of iron deficiency and iron deficiency anemia from tenth donation and further should be considered.

There were a few studies that evaluated iron replacement protocol in blood donors.[16–18] Recently, Magnussen *et al*. have confirmed the effect of an iron replacement protocol (with 50 mg iron) on increasing activation time of low Hb blood donors.[17] Performing a future study for designing an iron replacement protocol in Iran is recommended.

## Conclusion

As a whole considering this study’s results, iron deficiency, and iron deficiency anemia were seen among 2.3% and 2.14% of regular donors respectively, and a significant relationship was seen between iron deficiency indicator indexes, and higher than five instances of donation, and also between iron deficiency, iron deficiency anemia, and higher than ten instances of donation, so that this result correlated with previous study results.[1,7,8,10,13,15–18] Therefore donors between fifth donation and tenth donation demand especial care in primary steps of examination before donation, and usage of ferritin evaluation as a more appropriate index for iron deficiency screening and replacement of iron stores in these donors is recommended.
